# Trends and characteristics of child non-vaccination in Guinea from 1999 to 2018

**DOI:** 10.3389/fpubh.2025.1725647

**Published:** 2026-01-12

**Authors:** Fatoumata Cherif, Dimai Ouo Kpamy, Sory Conde, Alexandre Delamou, Fodé Amara Traore, Seydou Doumbia, Peter John Winch, Abdoulaye Toure, Alioune Camara, Gbawa Camara, Diao Cisse

**Affiliations:** 1 National Agency for Health Security, Conakry, Guinea; 2 Faculty of Health Sciences and Technology, Gamal Abdel Nasser University of Conakry, Conakry, Guinea; 3 African Center of Excellence for Prevention and Control of Communicable Diseases, Conakry, Guinea; 4 University of Science, Technology, and Techniques, Bamako, Mali; 5 Johns Hopkins School of Public Health, Baltimore, MD, United States; 6 Research and Training Center in Infectiology, Conakry, Guinea; 7 National Malaria Control Program, Conakry, Guinea; 8 Doctors Without Borders (MSF), Brussels, Belgium

**Keywords:** characteristics, children, Guinea, non-vaccination, trends

## Abstract

**Introduction:**

The absence of vaccination among children remains a significant public health challenge in Guinea. The objective of this study was to describe the trends and profiles of unvaccinated children in Guinea in 1999, 2012, and 2018.

**Methods and materials:**

This was a descriptive cross-sectional study conducted from July to December 2024, focusing on children aged 12 to 24 months, as well as their mothers, using secondary data from the 1999, 2012, and 2018 Demographic and Health Surveys, DHIS2 databases, SAP (Excel), and measles laboratory registers (2012–2018). All databases were cleaned, merged, and validated to avoid duplicates or data entry errors, and the information was handled confidentially.

**Results:**

Between 1999 and 2018, unvaccinated children were mostly male (52 to 53%) and resided in rural areas (87 to 93%). Firstborn children (up to 90%) and mothers with low levels of education (88 to 96% had no schooling) were the most represented. Access to information was very limited, with 98 to 100% of mothers having no access to printed media, 47 to 58% never listening to the radio, and 78% never watching television. In 2018, 60% of mothers had not attended any prenatal consultations, and 80% of deliveries took place at home. The majority of confirmed measles cases affected unvaccinated children (85%), more than half of whom (52%) were aged 0 to 2 years. The geographic distribution mainly corresponded to certain regions with high non-vaccination rates, including Middle and Upper Guinea. Although the Nzérékoré region reported a lower number of unvaccinated children compared to other regions, it paradoxically had the highest number of measles cases in 2012, accounting for 67% of reported cases.

**Conclusion:**

Our findings show that non-vaccination primarily affects children in rural areas, with uneducated, poorly informed mothers and limited access to healthcare. A targeted, community-based approach is essential to improve vaccination coverage and prevent epidemics.

## Introduction

Vaccination saves children’s lives; it has helped control many infectious diseases in developed countries but remains essential in developing countries where these infections continue to cause numerous child deaths (1, 2). In 2020, the number of children who did not receive routine vaccination was estimated at 22.7 million, an increase of 19.5% compared to 2019. The number of children completely unvaccinated rose from 13.6 million in 2019 to 17.1 million in 2020 (3). Over the past 10 years, vaccination coverage has stopped progressing in many countries. This trend worsened with the COVID-19 pandemic, which severely disrupted vaccination services worldwide, causing delays in campaigns and insufficient access to routine vaccination (3). The decline in vaccination reveals the impact of inequalities: unvaccinated children often grow up in families where mothers, who are uneducated and have little influence in decision-making, face fragile healthcare systems. The COVID-19 pandemic only worsened these difficulties by diverting resources and exposing persistent shortages in staff, equipment, and access to essential care (4). Worldwide, it is estimated that between 2019 and 2021, 67 million children missed their routine vaccinations either partially or completely. Enormous inequalities exist between regions, with the highest prevalence of children who have not received any doses in African regions, but inequalities also exist within regions (3, 4). The analysis of demographic and health survey data from 34 countries in sub-Saharan Africa shows that vaccination coverage remains uneven, with high rates of non-vaccination in certain regions. This study presents results comparable to those observed in Guinea, where vaccination coverage has shown a tendency to stagnate, with significant geographical disparities (5) In West and Central Africa, this concerns 19.5 million children (4), Of which nearly 3.1 million are in Nigeria, accounting for about 14% of the regional total (6). In Guinea, according to the 2018 Demographic and Health Survey (DHS), the percentage of children who did not receive any vaccines from the Expanded Program on Immunization (EPI) is 22% for children aged 12–23 months (7). After a slight increase in vaccination coverage for children in Guinea between 1999 and 2005 (from 32 to 37%), the situation remained stable until 2012 and then significantly deteriorated in 2018, with only 24% of children having received all the basic vaccines, thus a lower level than in 1999. At the same time, the proportion of unvaccinated children, which had decreased until 2012, once again increased, reaching a level in 2018 close to that of 1999 (7). In 2021, more than 281,000 children were under-immunized in Guinea, including nearly 192,000 who had not received any doses of the pentavalent vaccine. The majority of these zero-dose children were located in densely populated areas, particularly in the health districts of Siguiri and Boké (8).

To reverse this concerning increase in the number of unvaccinated children, it is important to identify who they are, where they live, and the reasons why they remain outside the vaccination system, with the aim of developing targeted strategies to reach them and protect them against vaccine-preventable diseases.

The objective of this study was to describe the trends and profiles of unvaccinated children in Guinea in 1999, 2012, and 2018.

## Study methods

### Study framework

The Republic of Guinea served as the study framework. It is located in the southwestern part of West Africa and covers an area of 245,857 km^2^. It is a coastal country with 300 km of Atlantic coastline, halfway between the equator and the Tropic of Cancer. It is bordered to the west by the Atlantic Ocean, to the south by Sierra Leone and Liberia; to the east by Côte d’Ivoire and Mali; and to the north by Guinea-Bissau, Senegal, and Mali. It is characterized by a climate with two seasons (dry and rainy), with an average duration of 6 months, and the rainy season can last up to 9 months in the southeast.

**Figure 1:**
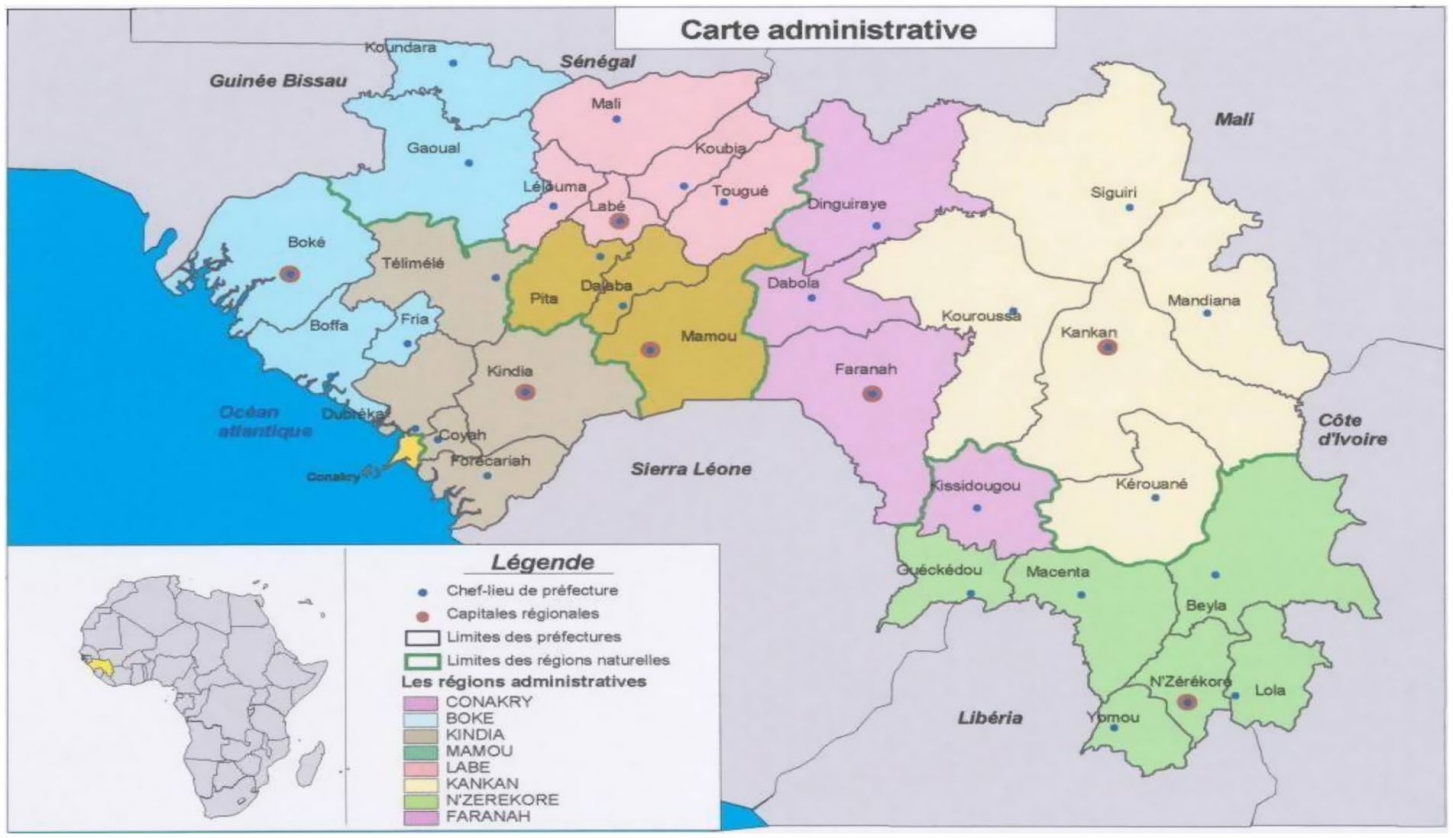
Administrative map of Guinea (8). Source: All these administrative entities and local governments fall under the Ministry of Territorial Administration and Decentralization. The natural regions are Lower Guinea, Middle Guinea, Upper Guinea, and Forest Guinea.

From an administrative standpoint, Guinea is divided into seven administrative regions: Boké, Faranah, Kankan, Kindia, Labé, Mamou, and Nzérékoré, with the capital Conakry having a special status. Each region is governed by a Governor. The administrative regions are subdivided into 33 prefectures across the country. Each prefecture is further divided into sub-prefectures administratively, and urban and rural communes as part of decentralization. There are a total of 343 urban and rural communes, including the 5 communes of Conakry. The communes are divided into districts and neighborhoods. All these administrative entities and local governments fall under the Ministry of Territorial Administration and Decentralization. The natural regions are Lower Guinea, Middle Guinea, Upper Guinea, and Forest Guinea (8) (see Figure 1).

### Study type and duration

This was a descriptive cross-sectional study based on the analysis of secondary data extracted from the Demographic and Health Surveys (DHS) conducted in 1999, 2012, and 2018, the DHIS2 epidemiological surveillance databases, laboratory registers covering the period from 2012 to 2018, and SAP (Excel). Data cleaning and analysis were conducted over a period of 6 months. The objective of the Demographic and Health Survey (DHS) was to collect data on maternal and child health indicators, health practices, and access to healthcare services within the Guinean population, in order to inform public health policies and improve the planning of health interventions. Access to these databases was officially authorized by the relevant authorities.

### Study population, inclusion criteria, and sampling

The study focused on all unvaccinated children aged 12–24 months and their mothers listed in the DHS databases, as well as all suspected and confirmed cases of measles recorded during the study period. Inclusion was done exhaustively, and records with incomplete data or missing essential information were excluded from the final analysis.

### Variables

The study variables include individual, social, and health characteristics such as the child’s age and sex, birth order, region, mother’s age and education level, media access, religion, ethnic affiliation, number of prenatal consultations (PNC), place of delivery, the child’s vaccination status, as well as suspected and confirmed cases of measles.

### Data collection, validation, and analysis procedures

Data collection was carried out using a structured protocol. The ‘women’ questionnaire from the DHS served as the main tool. The entire database was cleaned, merged, and validated to eliminate duplicates and entry errors. All information was treated confidentially. The analysis was based on a two-stage stratified random sampling, following the DHS methodology. At the first stage, clusters or enumeration areas were selected nationwide from the list of general population and housing census areas. To do this, the country was divided into 8 study domains, corresponding to the 7 administrative regions and the capital, Conakry. In each study domain (except for the city of Conakry, which has no rural area), two strata were formed: the urban stratum and the rural stratum (7). The analysis included all households with unvaccinated children aged 12 to 24 months. It was carried out using R software, version 4.5.1. Statistical analyses were performed using Stata software (version 11) and Epi Info (version 11). Qualitative variables were presented as frequencies and proportions, while quantitative variables were expressed as median and interquartile range (IQR). Characteristics related to non-vaccination were assessed based on the description of data collected from mothers and children. The analysis of non-vaccination trends involved comparing vaccination coverage rates for children between the years 1999, 2012, and 2018 across the different administrative regions of Guinea. A spatial and temporal analysis highlighted regional variations in non-vaccination.

## Results

Table 1 shows that between 1999 and 2018, the gender distribution of unvaccinated children remained relatively stable, with a slight male predominance, representing 52 to 53%. Regarding birth order, the majority of children were first-born (86 to 90%), followed by second-born children, representing approximately 10 to 13%. The median age of mothers remained stable around 28 years (IQR: 22–35 years), with the most represented age group being 25 to 29 years (25 to 28%), followed by 20 to 24 years (20 to 23%) and 30 to 34 years (18 to 22%). Religiously, 91 to 95% of mothers were Muslim, while 3.2 to 4.6% were Christian. Ethnically, the majority of mothers were Fulani (38 to 61%), followed by Malinké (25 to 46%) and Susu (5.7 to 9.6%). In 2018, 88% of unvaccinated children lived in rural areas, compared to 12% in urban areas. The maternal education level remained very low, with 88% of mothers being uneducated and only 3.2% having reached secondary school level. Regarding access to information and media, 98 to 100% of mothers had no access to the written press, 47 to 58% never listened to the radio, and 78 to 79% never watched television. Only 10 to 12% claimed to have access to television at least once a week. As for the use of healthcare services, 47 to 62% of mothers did not attend prenatal consultations, and only 12 to 21% had more than three visits. Regarding the place of delivery, 80 to 96% of births took place at home.

**Table 1:** Distribution of unvaccinated children and their mothers in Guinea (1999–2018) according to socio-demographic characteristics, access to healthcare, and information.

Characteristics	*N*	1999 *N* = 370^1^	2012 *N* = 219^1^	2018 *N* = 374^1^
Sex	963			
Male		196 (53%)	112 (51%)	195 (52%)
Female		174 (47%)	107 (49%)	179 (48%)
Birth order	963			
1		332 (90%)	198 (90%)	323 (86%)
2		37 (10%)	21 (9.6%)	50 (13%)
3		0 (0%)	0 (0%)	1 (0.3%)
4		1 (0.3%)	0 (0%)	0 (0%)
Median (Q1, Q3); *n* (%)		28 (23, 34)	28 (22, 34)	28 (24, 35)
Age	963			
Mother’s age group	963			
15–19		24 (6.5%)	22 (10%)	29 (7.8%)
20–24		85 (23%)	47 (21%)	74 (20%)
25–29		100 (27%)	54 (25%)	104 (28%)
30–34		70 (19%)	48 (22%)	69 (18%)
35–39		54 (15%)	29 (13%)	58 (16%)
40–44		29 (7.8%)	10 (4.6%)	27 (7.2%)
45–49		8 (2.2%)	9 (4.1%)	13 (3.5%)
Religion of mothers	959			
Muslim		334 (91%)	207 (95%)	355 (95%)
Christian		17 (4.6%)	7 (3.2%)	17 (4.5%)
Animist		4 (1.1%)	0 (0%)	0 (0%)
No religion		11 (3.0%)	5 (2.3%)	2 (0.5%)
Other		0 (0%)	0 (0%)	0 (0%)
Unknown		4	0	0
Ethnicity of mothers	963			
Soussou		21 (5.7%)	21 (9.6%)	36 (9.6%)
Peulh		142 (38%)	125 (57%)	228 (61%)
Malinké		171 (46%)	59 (27%)	92 (25%)
Kissi		10 (2.7%)	3 (1.4%)	4 (1.1%)
Toma		4 (1.1%)	0 (0%)	0 (0%)
Guerzé		21 (5.7%)	9 (4.1%)	13 (3.5%)
Other		1 (0.3%)	2 (0.9%)	0 (0%)
Foreigner		0 (0%)	0 (0%)	1 (0.3%)
Residence	963			
Urban		26 (7.0%)	29 (13%)	46 (12%)
Rural		344 (93%)	190 (87%)	328 (88%)
Mother’s education level	963			
Uneducated		355 (96%)	196 (89%)	330 (88%)
Primary		13 (3.5%)	18 (8.2%)	28 (7.5%)
Secondary		2 (0.5%)	5 (2.3%)	12 (3.2%)
University		0 (0%)	0 (0%)	4 (1.1%)
Newspaper	592			
None		0 (NA%)	218 (100%)	368 (98%)
Less than once a week		0 (NA%)	0 (0%)	2 (0.5%)
At least once a week		0 (NA%)	0 (0%)	4 (1.1%)
Unknown		370	1	0
Radio	593			
None		0 (NA%)	103 (47%)	218 (58%)
Less than once a week		0 (NA%)	62 (28%)	79 (21%)
At least once a week		0 (NA%)	54 (25%)	77 (21%)
Unknown		370	0	0
Television	593			
None		0 (NA%)	172 (79%)	293 (78%)
Less than once a week		0 (NA%)	21 (9.6%)	43 (11%)
At least once a week		0 (NA%)	26 (12%)	38 (10%)
Unknown		370	0	0
Number of prenatal consultations (PNC)	885			
No prenatal visits		225 (62%)	94 (47%)	193 (60%)
1		30 (8.2%)	19 (9.6%)	31 (9.6%)
More than 3 prenatal consultations (PNC)		59 (16%)	41 (21%)	38 (12%)
2–3		50 (14%)	44 (22%)	59 (18%)
Do not know		0 (0%)	0 (0%)	2 (0.6%)
Unknown		6	21	51
Place of delivery	963			
Home		357 (96%)	187 (85%)	301 (80%)
Public sector		10 (2.7%)	28 (13%)	63 (17%)
Private sector		3 (0.8%)	4 (1.8%)	10 (2.7%)

^1^*N*, Sample size; NA, Not applicable; PNC, Prenatal Consultations.

**Figure 2:**
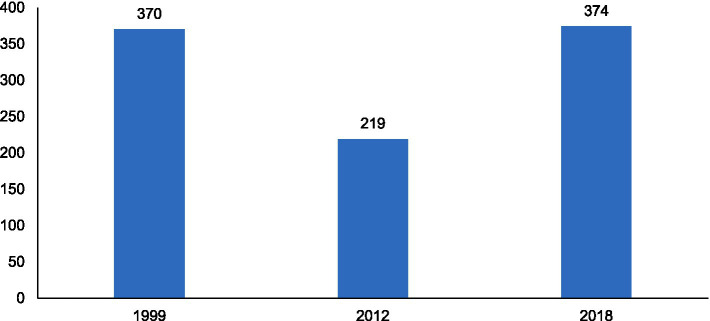
Analysis of trends in unvaccinated children in 1999, 2012, and 2018.

**MAP 1:**
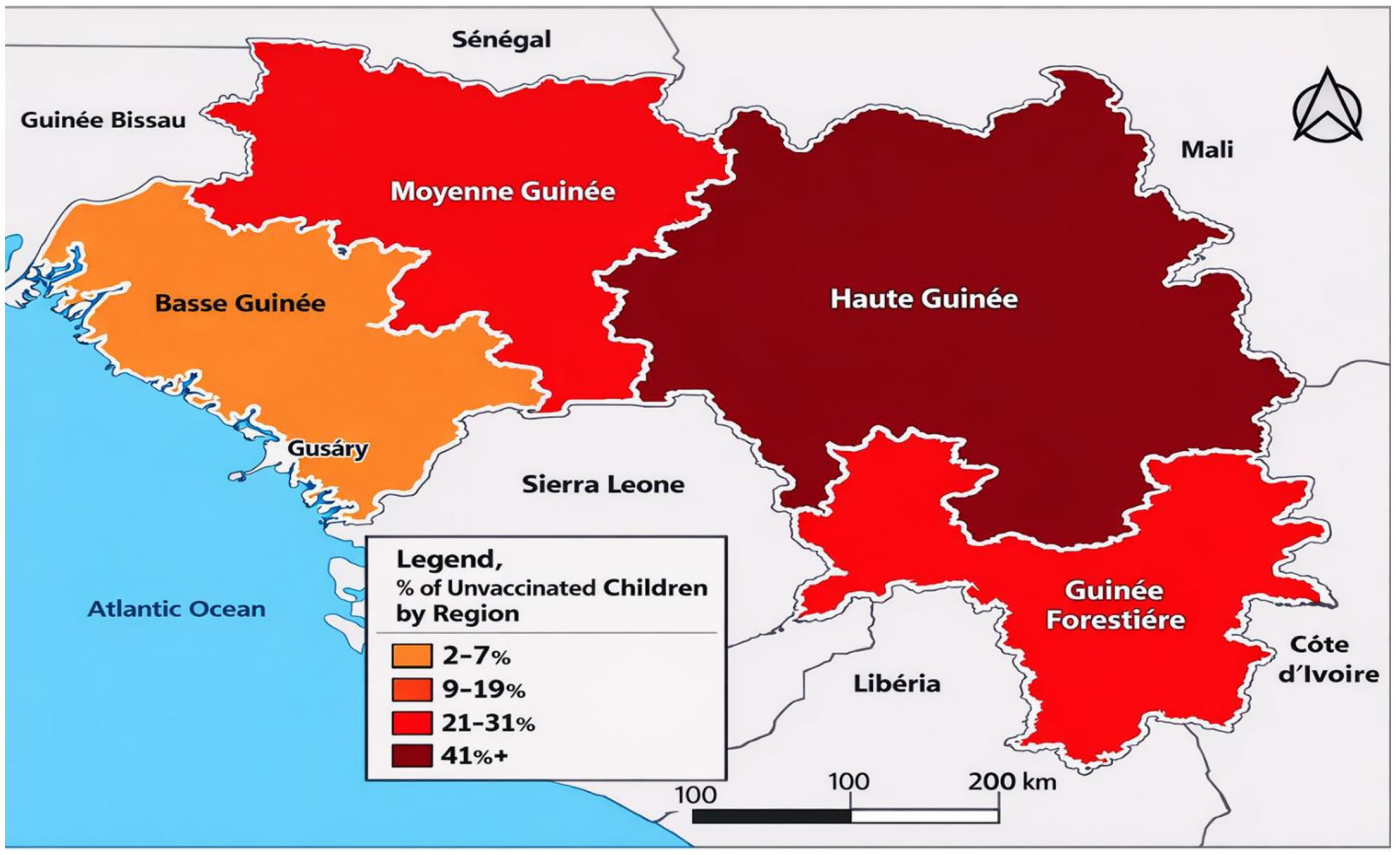
Situation of unvaccinated children by region, DHS Guinea 1999. Source: Demographic and Health Survey (DHS) 1999, Ministry of Health, Republic of Guinea.

**MAP 2:**
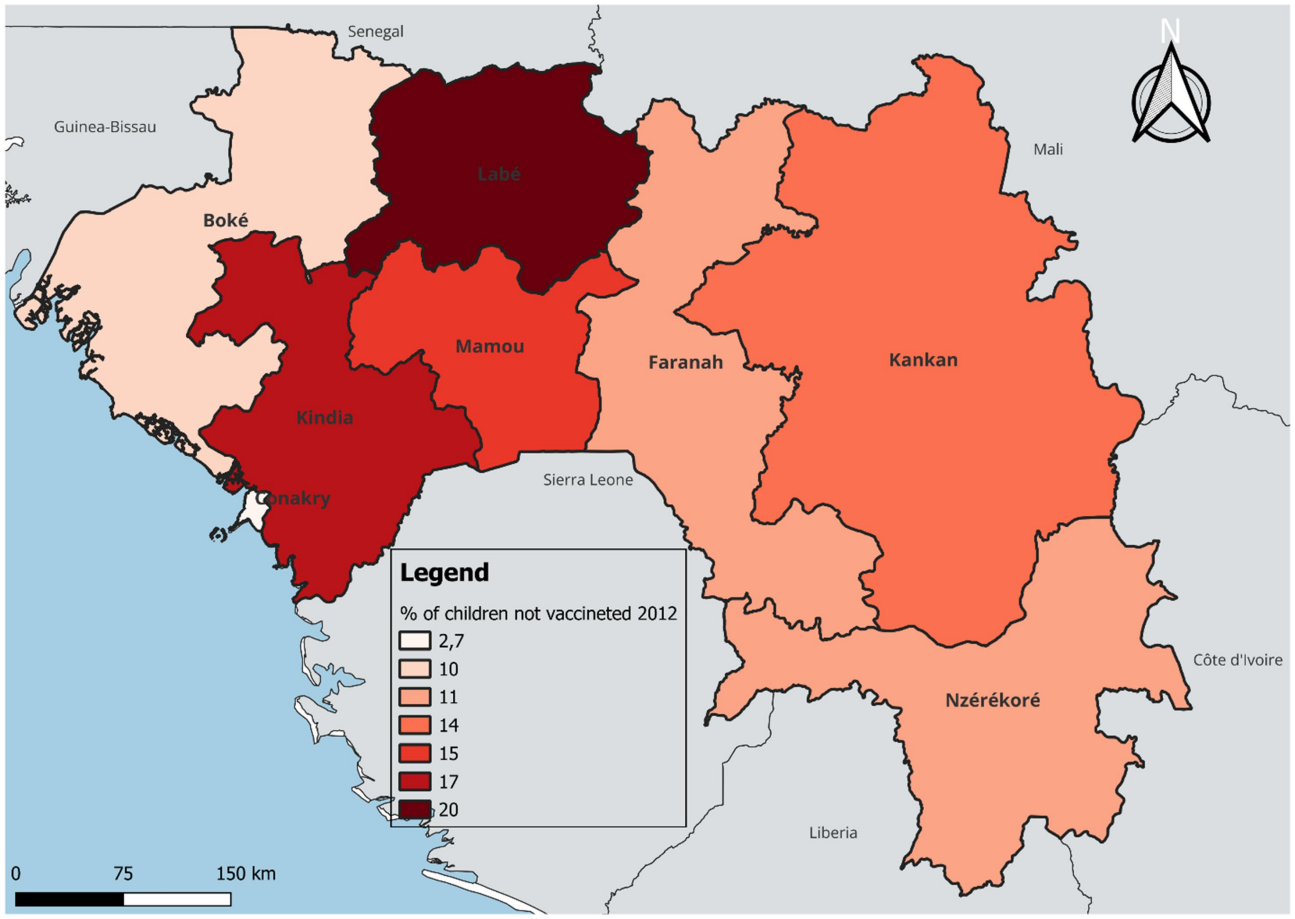
Situation of unvaccinated children by region, DHS Guinea 2012. Source: Demographic and Health Survey (DHS) 1999, Ministry of Health, Republic of Guinea.

**MAP 3:**
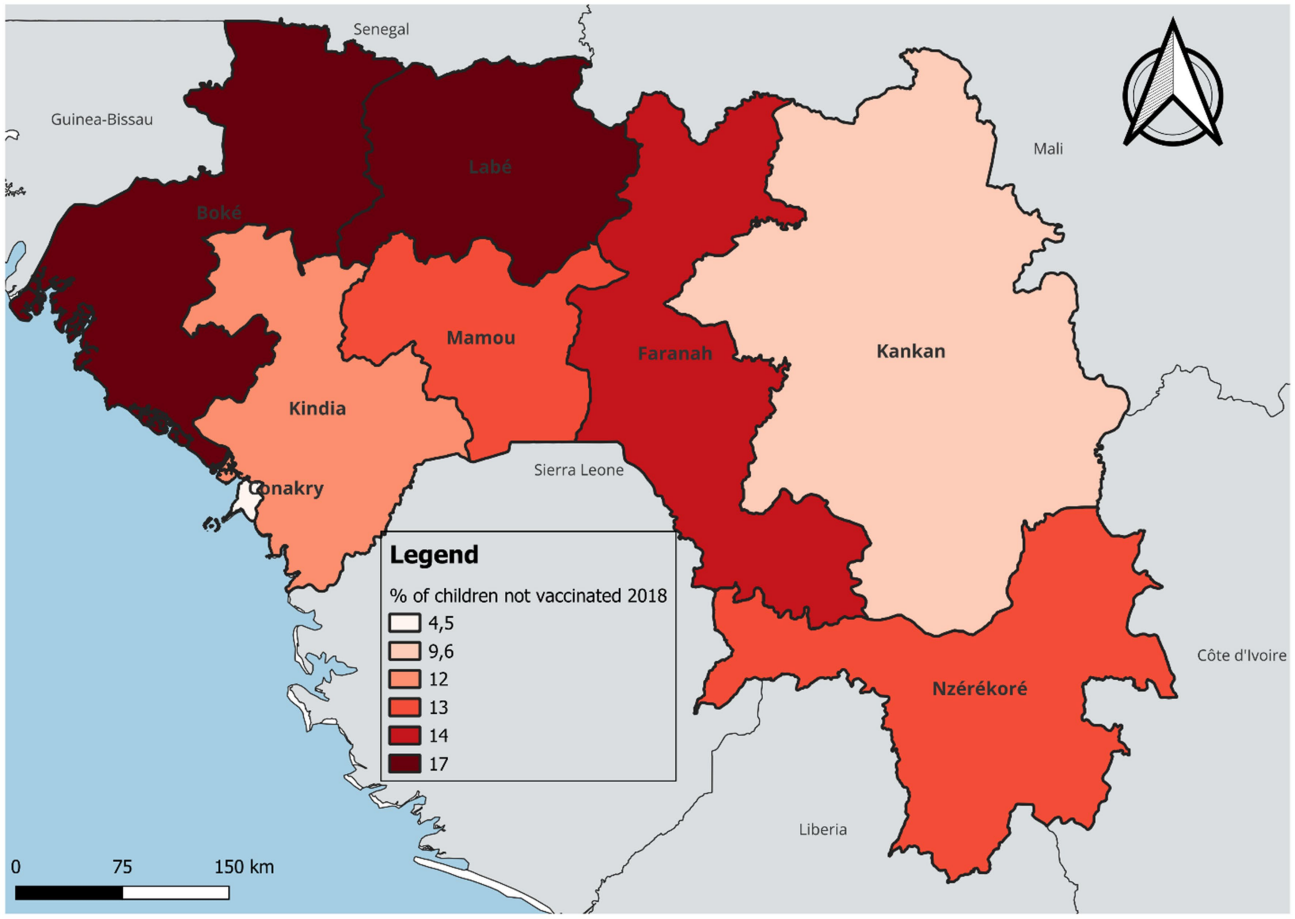
Situation of unvaccinated children by region, DHS Guinea 2018. Source: Demographic and Health Survey (DHS) 2018, Ministry of Health, Republic of Guinea.

**MAP 4:**
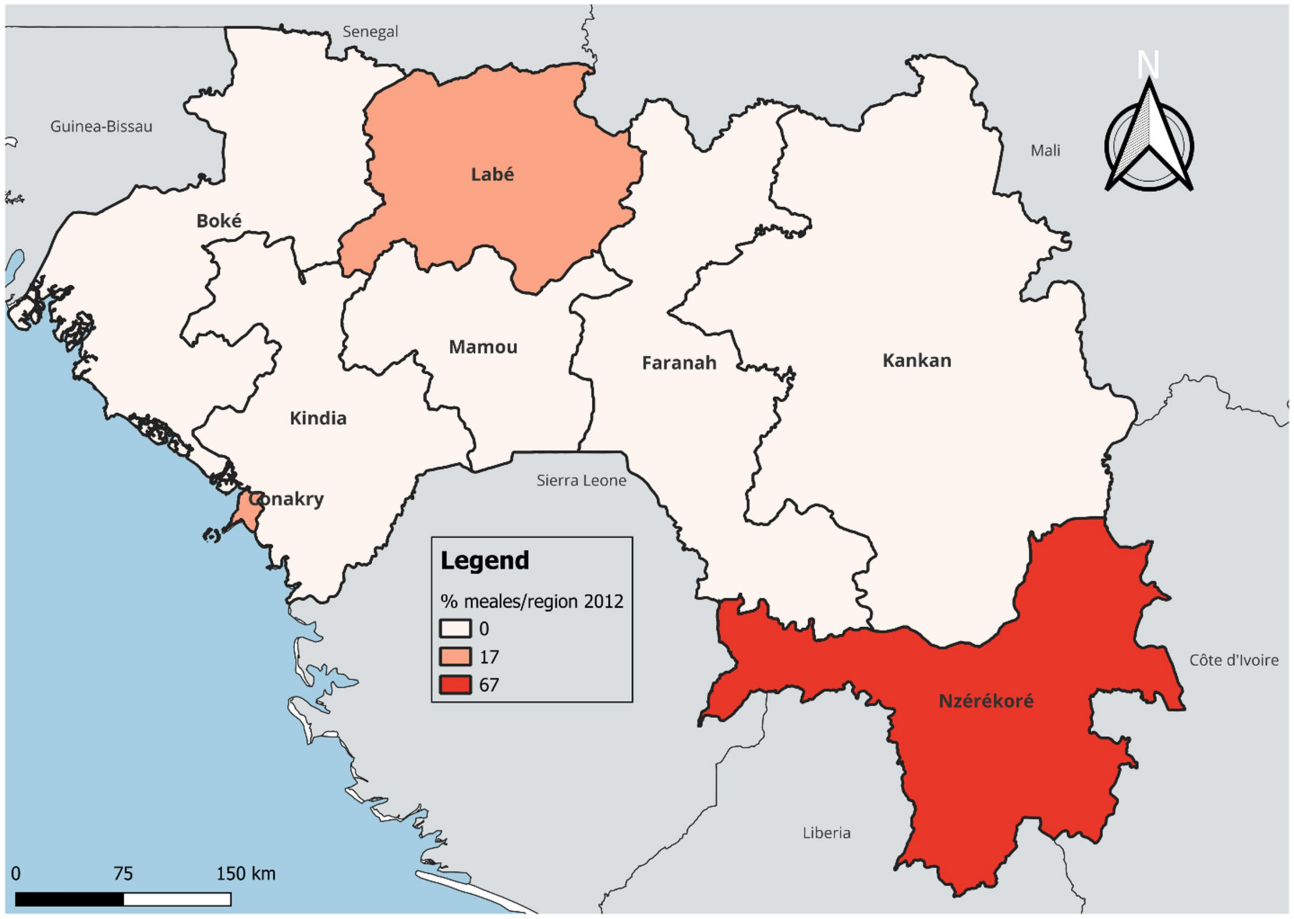
Situation of measles cases by region, Guinea 2012. Source: Demographic and Health Survey (DHS) 2012, Ministry of Health, Republic of Guinea.

**MAP 5:**
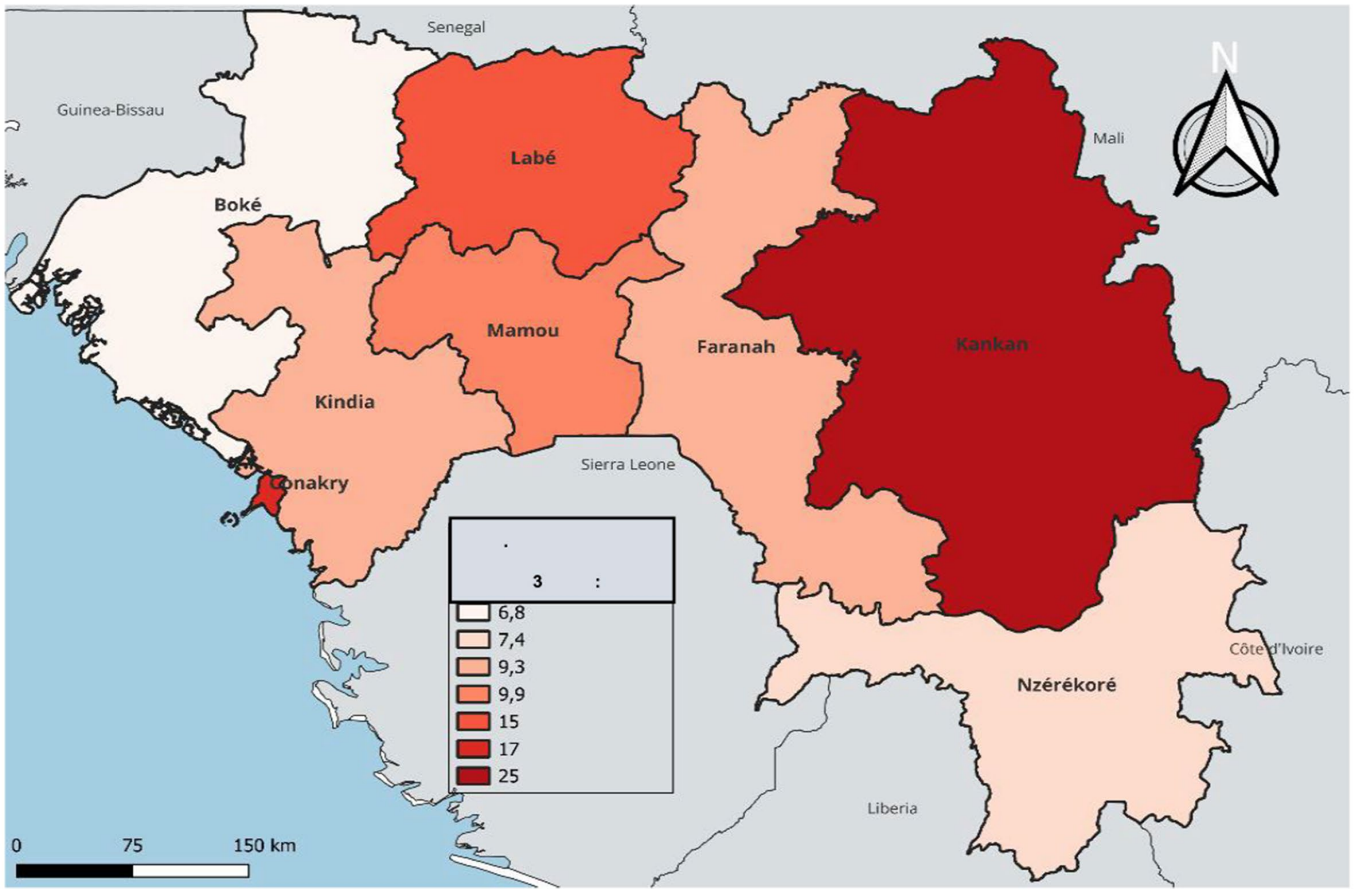
Situation of measles cases by region, Guinea 2018. Source: Demographic and Health Survey (DHS) 2018, Ministry of Health, Republic of Guinea.

In terms of temporal and regional trends over the years studied, the number of unvaccinated children remained high, with significant regional disparities. Middle Guinea and Upper Guinea had the highest proportions, particularly in rural areas (Figure 2, Maps 1–3). Regarding measles cases and their link to vaccination, it was observed that in 2018, the majority of confirmed measles cases involved unvaccinated children in 85% of cases, with more than half (52%) being aged 0 to 2 years. Their geographic distribution mainly corresponded to regions with high non-vaccination rates, notably Middle and Upper Guinea. Although the N’zérékoré region reported a lower number of unvaccinated children than other regions, it paradoxically had the highest number of measles cases in 2012, with 67% of measles cases (Maps 4, 5, Table 2).

**Table 2:** Distribution of 336 confirmed measles cases, 2012, 2018, Guinea according to sociodemographic characteristics and vaccination status.

Characteristics	2012 *N* = 12^1^	2018 *N* = 324^1^
Tranche d’âge		
0–2 years	10 (83%)	170 (52%)
2–4 years	0 (0%)	98 (30%)
4 years and older	2 (17%)	56 (17%)
Sex		
Male	6 (50%)	158 (49%)
Female	6 (50%)	166 (51%)
Regions		
Boké	0 (0%)	22 (6.8%)
Conakry	2 (17%)	56 (17%)
Faranah	0 (0%)	30 (9.3%)
Kankan	0 (0%)	80 (25%)
Kindia	0 (0%)	30 (9.3%)
Labé	2 (17%)	50 (15%)
Mamou	0 (0%)	32 (9.9%)
N’Nzérékoré	8 (67%)	24 (7.4%)
Vaccination		
No	12 (100%)	274 (85%)
Yes	0 (0%)	50 (15%)

^1^*n* (%); *N*, effectifs.

## Discussion

Vaccination is an effective means of preventing certain childhood diseases when administered at the appropriate time and followed up regularly. Our study revealed that the non-vaccination rate was particularly high among children of poorly educated mothers living in rural areas. In Sub-Saharan Africa, children of parents who have not received formal education are at a higher risk of being unvaccinated compared to those whose mothers or fathers have reached secondary or higher education levels (9). In a Senegalese study where full vaccination coverage was estimated at 62.8% among children aged 12 to 23 months, it was demonstrated that the low level of maternal education reduces the likelihood of children being vaccinated (10). A study conducted in Ghana in 2024 found that the completeness of vaccination status was significantly associated with the mother’s secondary education level (ORaj = 2.60) and tertiary education level (ORaj = 3.98), as well as with the proximity to a healthcare facility (11). However, in Burkina Faso in 2017, maternal factors, gender, and place of birth had no influence on vaccination coverage (12). Uneducated mothers face difficulties in understanding the importance of vaccines, following the vaccination schedule, or accessing information campaigns. In rural areas, distance, road conditions, and lack of infrastructure further exacerbate these access challenges to healthcare, highlighting the crucial importance of maternal education for child health (12–17). In our study, almost all mothers had no access to newspapers, two-thirds did not watch television, and more than half never listened to the radio. However, according to a study conducted in 24 Sub-Saharan African countries, maternal access to media reduced the probability of a child being unvaccinated by 6% (OR 0.94, 95% CI: 0.94 to 0.98) (9, 18). Hence the importance of access to information to ensure the completeness of children’s vaccination status. This also suggests that disadvantaged households are often the ones that benefit the least from awareness campaigns. In our study, we observed a high rate of lack of prenatal consultation. Similar results were reported in a meta-analysis of data from several countries, which showed that the absence of prenatal consultation increased the risk of non-vaccination (ORaj = 2.6; 95% CI: 1.4–5.1). Another multi-country study found that children whose mothers had never had prenatal follow-up were less likely to receive all recommended vaccinations (ORaj = 0.76; 95% CI: 0.71–0.82) (19). The analysis of demographic and health survey data from 34 countries in sub-Saharan Africa shows that vaccination coverage remains uneven, with high rates of non-vaccination in certain regions. This study presents results comparable to those observed in Guinea, where vaccination coverage has shown a tendency to stagnate, with significant geographical disparities (5). This highlights an increased risk of missing vaccination appointments. However, these consultations represent crucial opportunities for exchange, awareness, and education for expectant mothers.

From the data studied, the number of unvaccinated children remains high, with a significant proportion observed in Middle and Upper Guinea, predominantly in rural areas. A qualitative study conducted in Conakry emphasizes that vaccinations were largely perceived positively by the population, and their preventive benefits were well recognized (20). Similar observations have been reported in Ethiopia, where children living in rural areas were significantly more exposed to the risk of non-vaccination or incomplete vaccination compared to those in urban areas (21). Similarly, analyses from the Demographic and Health Surveys regularly highlight the weakness of vaccination coverage in Upper and Middle Guinea compared to urban areas (22). Hence the need to take into account social realities, community perceptions, and even trust between healthcare providers and patients in the planning of vaccination strategies. In our series, we also noted that measles occurred in regions with a high proportion of unvaccinated children. This is consistent with literature data showing that the occurrence of epidemics indicates suboptimal vaccination coverage (23).

However, we observed a paradoxical phenomenon in the N’zérékoré region, which had fewer unvaccinated children but was the most affected by measles in 2012. The literature has shown that even with relatively high vaccination coverage, measles outbreaks can occur if immunity distribution is uneven or if pockets of vulnerable populations persist (24). This situation in our context suggests that other contextual factors, yet to be explored, may have favored the spread of the epidemic in this locality.

Knowing that the use of secondary data has limitations, including reporting biases or the lack of certain contextual variables, a rigorous process of cleaning, validation, and anonymization was carried out to reduce these biases and ensure the reliability of the results.

This study has some limitations related to the use of secondary data, including reporting biases and missing information. Furthermore, the sample used in the study does not uniformly represent all regions of Guinea, which may introduce geographical biases in the analysis of non-vaccination trends. Additionally, the available data does not allow for the exploration of certain contextual factors, such as local community perceptions of vaccines, which may influence adherence to vaccination programs. The 1999 survey, conducted in the four natural regions, and the surveys of 2012 and 2018, conducted in the administrative regions, limited the comparison of the proportions of children between the different regions. Additionally, the absence of the 1999 measles database was due to a surveillance system that was still underdeveloped in the country at that time.

### Policy implication of findings

The results of this study highlight the importance of improving access to education for mothers, particularly in rural areas, in order to reduce non-vaccination rates. Political interventions should aim to strengthen maternal and child health services by increasing access to prenatal consultations and postnatal care. It is also crucial to adopt targeted communication strategies, using local media, to raise awareness among vulnerable communities about the benefits of vaccination and the need to adhere to the vaccination schedule. It would also be relevant to conduct a study in the future on the factors associated with non-vaccination among children of less educated mothers and those living in rural areas.

## Conclusion

Our study revealed that non-vaccination mainly affects children living in rural areas, whose mothers are poorly educated, misinformed, and have very limited access to healthcare. This situation particularly exposes children to the risks of vaccine-preventable diseases, such as measles. We will recommend targeted actions in the regions of Upper Guinea and Middle Guinea, where non-vaccination rates are the highest. We will also propose interventions to improve healthcare access in rural areas and strengthen awareness campaigns, focusing on maternal education and healthcare accessibility. The engagement of local communities, along with the effective use of local media, is a key lever to overcome these persistent barriers and ensure that every child has equitable access to vaccination.

## Data Availability

The datasets presented in this study can be found in online repositories. The names of the repository/repositories and accession number(s) can be found below: Données EDS.
